# Associations between vitamin B6 and vitamin B12 intake and lung function: A cross-sectional study based on NHANES 2007 to 2012

**DOI:** 10.1097/MD.0000000000049118

**Published:** 2026-06-05

**Authors:** Wuzhen Wang, Yingying Gu

**Affiliations:** a School of Clinical Medicine, Jiangxi University of Chinese Medicine, Nanchang, Jiangxi, China; b School of Nursing, Jiangxi Medical College, Nanchang University, Nanchang, Jiangxi, China.

**Keywords:** lung function, NHANES, nutrition, vitamin B12, vitamin B6

## Abstract

Although the benefits of enhanced nutrition on respiratory health are widely recognized, the specific effects of vitamins B6 and B12 on lung function are not fully understood. This study aimed to investigate the potential association between vitamin B6 and B12 intake and lung function. This is a cross-sectional study using data from the National Health and Nutrition Examination Survey from 2007 to 2012. The study assessed the pulmonary function of the subjects by measuring forced expiratory volume in the first second (FEV_1_), forced vital capacity (FVC), and the FEV_1_/FVC ratio. In addition, stepwise multiple linear regression, logistic regression modeling, and other analytical methods were used in this study in order to investigate the correlation between vitamin B6 and B12 intake and lung function. A total of 9648 participants were included in this study, and after adjusting for relevant confounders, linear regression analyses showed that for every 1 mg/day increase in vitamin B6 intake, there was an increase in FVC of 22 mL (*P* = .004). Meanwhile, for every 1 μmg/day increase in vitamin B12 intake, there was an increase of 2.2 mL (*P* = .006) in FEV_1_ and 3.8 mL (*P* < .001) in FVC. In addition, no association was found between vitamin B6 and B12 intake and the FEV_1_/FVC ratio. There was no gender difference in the effect of vitamin B6 and B12 intake on lung function, and there was a significant trend toward improvement in all relevant indicators with increasing intake. Weighted logistic regression analysis showed that no association was found between vitamin B6 and B12 intake and the risk of airway obstruction. In this regard, we think that higher intake levels of vitamins B6 and B12 may be associated with better lung health.

## 1. Introduction

Pulmonary diseases constitute a group of chronic conditions affecting millions globally and significantly impact overall mortality rates.^[1–3]^ Lung function serves not only as a crucial predictor of mortality in the general population but also as a key determinant of prognosis in patients with pulmonary diseases and is associated with various physiological changes occurring with aging.^[4,5]^ Consequently, effectively improving lung function has become a critical issue requiring resolution. Therefore, the present study utilized data from 3 cycles of the National Health and Nutrition Examination Survey (NHANES) conducted between 2007 and 2012 to investigate the relationship between vitamin B6 and B12 intake and lung function. This research seeks to provide new insights at the population level for promoting respiratory health through dietary interventions.

Existing research indicates that diet exerts protective effects against conditions such as heart disease and cancer.^[6]^ However, studies examining the relationship between diet and lung health remain relatively scarce, particularly regarding the impact of vitamins on lung function in both healthy individuals and patients with pulmonary diseases. As 2 crucial water-soluble vitamins, vitamins B6 and B12 may exert positive effects on lung function through mechanisms including reducing the risk of death,^[7]^ anti-inflammatory action, immune modulation, and optimization of cellular metabolism.^[8]^ For instance, vitamin B6 may alleviate type 2 inflammation by regulating interleukin‑33 homeostasis, thereby improving respiratory function.^[9]^ Meanwhile, vitamin B12 has demonstrated therapeutic adjuvant effects and safety in combination therapy for patients with non-small cell lung cancer.^[10,11]^

Although these biological mechanisms and clinical observations suggest potential benefits of vitamins B6 and B12 for lung health, current epidemiological evidence regarding the relationship between vitamin intake and lung function in the general healthy population remains limited and lacks systematic population-based assessments. Therefore, this study will utilize NHANES data to further analyze whether dietary intake of vitamins B6 and B12 correlates with pulmonary function indicators, thereby providing scientific evidence for future public health interventions aimed at preventing pulmonary diseases.

## 2. Materials and methods

### 2.1. Data source

This is a population-based cross-sectional study with data from the NHANES database managed by the National Center for Health Statistics (NCHS).^[12]^ NHANES is a study that uses a stratified, multistage design to comprehensively assess the health and nutritional status of adults and children in the United States through a combination of interviews, physical examinations, and laboratory tests. The study’s complex, multistage design ensures that the data collected and analyzed accurately reflect the health of the nation’s noninstitutionalized population in the United States. In addition, the NHANES database is freely available, has been approved by the NCHS Institutional Review Board, and all participants have signed informed consent forms.

### 2.2. Study population selection

This study included data from the 2007 to 2012 NHANES cycles for adults aged 20 years and older. Exclusion criteria included lack of data on forced expiratory volume in the first second (FEV_1_), forced vital capacity (FVC), airway obstruction status, vitamin B6 and B12 intake, body mass index (BMI), education level, or smoking status and those who had been diagnosed or told by a physician that they had a malignant tumor, asthma, chronic bronchitis, or emphysema. These past medical histories were determined using the NHANES “Health Status” questionnaire, which asked participants, “Has a doctor or other healthcare professional ever told you that you have asthma, cancer, chronic bronchitis, or emphysema?”

### 2.3. Study variables

#### 2.3.1. Assessment of daily intake of vitamins B6 and B12

Vitamin B6 and B12 intake data were collected based on a 24-hour dietary recall questionnaire from the NHANES database, which was evaluated through the United States Department of Agriculture’s Dietary Research Food and Nutrition Database.^[13]^ Each participant underwent 2 dietary recalls: the first was completed at the Mobile Examination Center visit, followed by a second recall via telephone follow-up 3 to 10 days later. Vitamin B6 and B12 intake recorded at the first dietary visit was used as the data analyzed in this study. According to the National Institutes of Health Office of Dietary Supplements, the recommended daily intake of vitamin B6 for men aged 51 years and older is 1.7 mg and for women aged 51 years and older is 1.5 mg, whereas, according to the US Food and Drug Administration reference intake, the recommended daily intake of vitamin B12 for adults is 2.4 μmg.^[14]^ This study uses the recommended intake of 1.7 mg/day for men aged 51 and over as the unified threshold. Although this is slightly higher than the requirement for adults under 50, it represents the highest recommended value for adults and can therefore conservatively ensure that the risk of inadequate intake is not underestimated across all age groups. Thus, vitamin B6 intake was categorized into 2 groups: below and above 1.7 mg, while the intake of vitamin B12 was categorized as <2.4 µmg versus >2.4 µmg.

#### 2.3.2. *FVC, FEV*_*1*_*, and their ratios (FEV*_*1*_*/FVC) were used as an assessment of lung function*

This study was based on the pre-bronchodilator spirometry method described in the NHANES database, which excludes the following: persons with current chest pain or difficulty expirating with exertion; persons who are receiving supplemental oxygen; persons who have had recent eye, thoracic, or abdominal surgery; persons who have had a recent myocardial infarction, stroke, exposure to tuberculosis, or signs of coughing up blood; adults with a personal history of retinal detachment or collapsed lung; and children with painful otitis media. Spirometry results were evaluated according to American Thoracic Society data quality standards.^[15]^ Examination data from pre-bronchodilator spirometry were used in the study, and FVC, FEV_1_, and their ratios (FEV_1_/FVC) were used as an assessment of lung function. Participants with FEV_1_/FVC <0.70 or below the lower limit of normal were determined to have airway obstruction according to the relevant study criteria.^[16]^

#### 2.3.3. Covariates

Sex, age, race, BMI, smoking and drinking status, poverty index, education level, sedentary time, dietary fiber intake, as well as diabetes mellitus, urinary protein and urinary creatinine levels, and the presence of a history of coronary heart disease, angina pectoris, congestive heart failure, hypertension, and stroke were considered potential confounders in this study.

BMI was categorized into 4 categories: ≤18.5 kg/m^2^, 18.5 to 24.9 kg/m^2^, 25.0 to 29.9 kg/m^2,^ and ≥30.0 kg/m^2^.

Smoking status was categorized into 3 groups: nonsmokers (<100 cigarettes in their lifetime), former smokers (more than 100 cigarettes in their lifetime but not currently smoking), and current smokers (more than 100 cigarettes in their lifetime and currently smoking).

Drinking frequency, as an indicator for assessing alcohol intake in the population, was categorized into 4 classes: never (<1 drink per year), low frequency (1–12 drinks per year), medium frequency (3–52 drinks per month), and high frequency (>52 drinks per year).

Sedentary time was assessed by asking participants how much time they spent watching television, playing video games, or using computers each day, which was categorized into 3 categories: <3 hours per day, 3 to 6 hours, and 6 hours or more.

Poverty is usually judged by whether the poverty index is <1.3, where groups with a poverty index of <1.3 are regarded as poor and those with a poverty index of more than 1.3 are categorized as non-poor.

Diagnoses of coronary heart disease, angina, congestive heart failure, hypertension, and stroke were made by asking participants, “Has a doctor or other health professional told you that you have any of these conditions?” to determine this.

The Oral Glucose Tolerance Test is a standardized test used to assess an individual’s ability to regulate blood glucose and pancreatic beta cell function. The test determines the presence of abnormal glucose metabolism by measuring blood glucose levels at various points in time after a prescribed amount of glucose has been ingested. According to the World Health Organization criteria, diabetes mellitus is diagnosed if a subject’s blood glucose level reaches or exceeds 11.1 mmol/L 2 hours after taking sugar in the Oral Glucose Tolerance Test.

Total dietary fiber intake was estimated based on data from the NHANES dietary interview. The interview assessed dietary fiber intake by asking participants about the types and amounts of food and beverages they had consumed in the 24 hours prior to the interview.

### 2.4. Statistical analysis

In all analyses, NCHS guidelines were followed, using the dietary day 1 sample weight (WTDRD1), the masked variance pseudo-stratum, and the masked variance pseudo-primary sampling unit provided by NHANES. These weights were developed to correct for complex survey designs (including oversampling), nonresponse bias, and post-stratification adjustments to match the Census Bureau’s total population counts. Each person in the sample is assigned a specific sample weight that reflects the overall number of people represented by that sample person. Therefore, WTDRD1 was selected as the analytic weight for the 1-day dietary recall data in this study.

In this study, the gtsummary package (developed by Daniel D. Sjoberg) was used to generate baseline tables and calculate sample sizes, proportions for categorical variables, and means for continuous variables. Continuous variables are presented as weighted percentages, including unweighted counts (n), percentages (%), and means.

To assess the associations between vitamin intake and lung function, we conducted analyses in 2 stages. First, we performed exploratory analyses using stepwise multivariable linear regression (backward selection based on the Akaike information criterion) to identify covariates independently associated with lung function parameters from a broad set of potential confounders. Additionally, we employed restricted cubic spline (RCS) regression with 4 knots placed at the 5th, 35th, 65th, and 95th percentiles of vitamin intake (using the rcs function from the R rms package [developed by Frank E Harrell Jr]) to visually assess and test for potential nonlinear relationships. The significance of nonlinearity was evaluated using the likelihood ratio test comparing models with and without the nonlinear terms. Second, and primarily, we constructed a series of prespecified multivariable regression models based on prior knowledge to rigorously test the association while controlling for defined sets of confounders. These models were the Crude (unadjusted) model; Model I (adjusted for sex, age, race, BMI, poverty-to-income ratio, and education level); Model II (further adjusted for smoking status and alcohol consumption frequency); and Model III (further adjusted for diabetes, total dietary fiber intake, urinary albumin, and urinary creatinine). We used weighted logistic regression models with the same prespecified adjustment sets to assess the association with airway obstruction (FEV_1_/FVC < 0.70). All analyses accounted for the complex survey design of NHANES using the survey package in R (version 4.2.1; developed by the International R Core Team, with major initial contributions from Ross Ihaka and Robert Gentleman), with a two-sided *P*-value < .05 considered statistically significant.

## 3. Results

### 3.1. Study population

**Figure 1.**
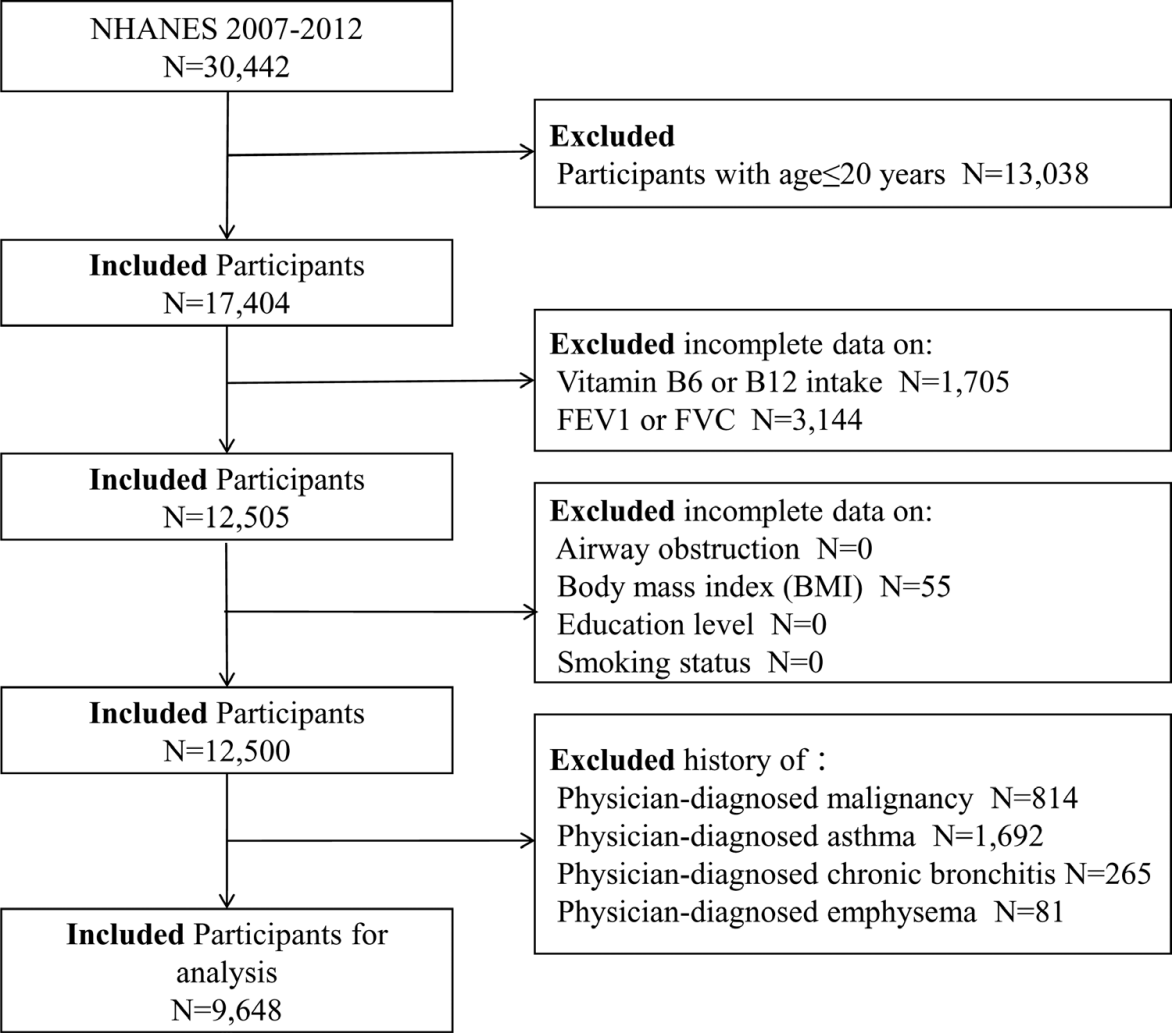
Study population. BMI = body mass index, FEV1 = forced expiratory volume in the first second, FVC = forced vital capacity, NHANES = National Health and Nutrition Examination Survey.

As shown in Figure 1, data from 3 consecutive cycles from 2007 to 2012 were selected for this study from the NHANES database, involving a total of 30,442 respondents. Of these participants, a total of 17,404 were older than 20 years. Subsequently, subjects with incomplete data on FEV_1_, FVC, airway obstruction status, vitamin B6 and B12 intake, BMI, level of education, smoking status, and those individuals who had been diagnosed or informed by a physician that they had a malignancy, asthma, chronic bronchitis, or emphysema were excluded. After these exclusions, the final number of subjects included in the analysis was 9648. The detailed participant selection process is shown in Figure 1.

### 3.2. Baseline characteristics of participants

Table 1 presents the distribution of baseline characteristics for the 9648 participants aged 20 years or older. The study presents the distribution of covariates stratified by gender. The cohort was more evenly distributed across age groups and had the highest proportion of non-Hispanic whites (66.1%) and 80.0% of those who were not poor. Mean FEV_1_ and FVC for all participants were 3227 and 4081 mL, respectively. By gender, mean FEV_1_ and FVC for male participants were 3823 and 4872 mL, respectively, whereas for females, they were 2758 and 3449 mL, respectively. The prevalence of airway obstruction was 11.0%. In terms of vitamin intake, the number of people who met the recommended intake of vitamin B6 and those who did not were roughly equal, but about a quarter of the population had a vitamin B12 intake below the recommended value. Smoking was a significant factor in lung function, with 56.0% of never smokers compared with 22.0% of current smokers. There were statistically significant differences between groups (*P* < .05) in race, age, BMI, smoking status, education level, frequency of alcohol consumption, dietary fiber intake, coronary heart disease, angina pectoris, urinary protein, and urinary creatinine.

**Table 1:** Baseline characteristics of study participants.

Study variables	Overall	Female	Male	*P*
	N = 9648	N = 4683	N = 4965	
Primary study variables				
Lung function				
FVC	4081 (3380, 4931)	3449 (2959, 3900)	4872 (4276, 5490)	**<.001**
FEV_1_	3227 (2642, 3889)	2758 (2314, 3167)	3823 (3286, 4336)	**<.001**
FEV_1_/FVC	0.80 (0.75, 0.84)	0.80 (0.76, 0.84)	0.79 (0.74, 0.83)	**<.001**
Airway obstruction				**<.001**
No (≥0.7)	8624 (89%)	4351 (92%)	4273 (87%)	
Yes (<0.7)	1024 (11%)	332 (8.1%)	692 (13%)	
Vitamin B6 (mg/d)				**<.001**
Above 1.7	5092 (56%)	1889 (42%)	3203 (68%)	
Below 1.7	4556 (44%)	2794 (58%)	1762 (32%)	
Vitamin B12 (μmg/d)				**<.001**
Above 2.4	6991 (75%)	3065 (68%)	3926 (82%)	
Below 2.4	2657 (25%)	1618 (32%)	1039 (18%)	
Demographic and socioeconomic				
Age				**.003**
20–30 yr	1914 (22%)	934 (21%)	980 (22%)	
30–40 yr	1991 (21%)	933 (19%)	1058 (23%)	
40–50 yr	1921 (23%)	948 (23%)	973 (22%)	
50–60 yr	1688 (19%)	806 (19%)	882 (18%)	
60–70 yr	1440 (11%)	706 (12%)	734 (10%)	
70–80 yr	694 (4.6%)	356 (5.3%)	338 (3.8%)	
Race				**.008**
Non-Hispanic White	3857 (66%)	1856 (66%)	2001 (66%)	
Non-Hispanic Black	2093 (11%)	1034 (12%)	1059 (11%)	
Mexican American	1763 (9.7%)	845 (8.9%)	918 (10%)	
Other Hispanic	1084 (6.0%)	548 (5.8%)	536 (6.2%)	
Other/multiracial	851 (6.8%)	400 (6.7%)	451 (6.9%)	
Poverty-income ratio				.100
Not poor (≥1.3)	6149 (80%)	2925 (79%)	3224 (80%)	
Poor (<1.3)	2689 (20%)	1362 (21%)	1327 (20%)	
Education level				**.045**
High school and above	7130 (83%)	3527 (84%)	3603 (82%)	
Never attended high school	2512 (17%)	1152 (16%)	1360 (18%)	
Anthropometric and lifestyle				
BMI				**<.001**
Normal (18.5–24.9)	2642 (29%)	1360 (33%)	1282 (25%)	
Obese (≥30)	3581 (35%)	1869 (35%)	1712 (35%)	
Overweight (25–29.9)	3299 (34%)	1376 (29%)	1923 (39%)	
Underweight (<18.5)	126 (1.4%)	78 (2.1%)	48 (0.7%)	
Smoking status				**<.001**
Current smoker	2100 (22%)	790 (18%)	1310 (25%)	
Former smoker	2069 (22%)	783 (19%)	1286 (26%)	
Never smoker	5474 (56%)	3109 (63%)	2365 (49%)	
Frequency of drinking alcohol				**.013**
Frequent (≥52 times/yr)	69 (0.9%)	16 (0.5%)	53 (1.3%)	
Low (1–12 times/yr)	6258 (83%)	2770 (83%)	3488 (82%)	
Moderate (13–52 times/yr)	158 (1.8%)	54 (1.3%)	104 (2.3%)	
Nondrinker (<1 times/yr)	1465 (15%)	695 (15%)	770 (14%)	
Sedentary time				.600
>6 h	2930 (35%)	1476 (34%)	1454 (35%)	
<3 h	2068 (17%)	1044 (17%)	1024 (16%)	
3–6 h	4641 (48%)	2160 (48%)	2481 (48%)	
Total dietary fiber intake	16 (10, 22)	14 (10, 20)	17 (11, 24)	**<.001**
Comorbidities				
Congestive heart failure	126 (1.0%)	48 (0.7%)	78 (1.2%)	.058
Coronary heart disease	205 (1.6%)	48 (0.7%)	157 (2.4%)	**<.001**
Angina pectoris	108 (0.9%)	36 (0.6%)	72 (1.2%)	**.024**
Stroke	171 (1.3%)	78 (1.1%)	93 (1.5%)	.300
Hypertension	2772 (25%)	1350 (24%)	1422 (26%)	.200
Diabetes				.400
Missing	6062 (65%)	2934 (64%)	3128 (66%)	
No (<11.1mmol/L)	3362 (34%)	1644 (34%)	1718 (33%)	
Yes (≥11.1mmol/L)	224 (1.6%)	105 (1.8%)	119 (1.5%)	
Laboratory measures				
Urine albumin	7 (4, 12)	6 (3, 12)	7 (4, 13)	**.003**
Urine Creatinine	110 (61, 168)	87 (47, 143)	131 (81, 185)	**<.001**

Bold values indicate statistically significant differences (*P* < .05).

BMI = body mass index, FEV1 = forced expiratory volume in the first second, FVC = forced vital capacity.

### 3.3. *The univariate correlation analysis between vitamin B6 and B12 intake and FEV*_*1*_*, FVC, and FEV*_*1*_*/FVC*

Table 2 summarizes the results of the one-way analysis of the association between lung function and vitamin B6 and B12 intake. We first used a one-way linear regression model to assess the association of vitamin B6 and B12 intake with FEV_1_, FVC, and FEV_1_/FVC. The analysis showed that vitamin B6 intake was significantly and positively correlated with FEV_1_ and FVC: for every 1 mg increase in vitamin B6, FEV_1_ and FVC increased by 142 mL (*P* < .001) and 191 mL (*P* < .001), respectively. For every 1 μmg increase in vitamin B12, FEV_1_, and FVC increased by 13 mL (*P* = .048) and 19 mL (*P* = .023), respectively. However, no significant association was found between vitamin B6 and B12 intake and the FEV_1_/FVC ratio.

**Table 2:** Unadjusted associations between vitamin B6 and B12 intake and lung function parameters.

	FEV_1_, mL		FVC, mL		FEV_1_/FVC (%)	
	Adjusted β (95% CI)	*P*	Adjusted β (95% CI)	*P*	Adjusted β (95% CI)	*P*
Vitamin B6 (mg/d)	142 (127, 157)	**<.001**	191 (172, 210)	**<.001**	0 (−0.01, 0.00)	.089
Below 1.7	Ref		443 (378, 507)		Ref	
Above 1.7	416 (361, 470)	**<.001**	552 (497, 607)	**<.001**	0 (−0.01, 0.00)	.140
Vitamin B12 (μmg/d)	13 (0.12, 25)	**.048**	19 (2.8, 35)	**.023**	0 (0.00, 0.00)	.087
Below 2.4	Ref		Ref		Ref	
Above 2.4	333 (277, 389)	**<.001**	443 (378, 507)	**<.001**	0 (−0.01, 0.00)	.093

Bold values indicate statistically significant differences (*P* < .05).

CI = confidence interval, FEV_1_ = forced expiratory volume in the first second.

### 3.4. *The correlation between vitamin B6 and B12 intake and FEV*_*1*_*, FVC, and FEV*_*1*_*/FVC in multivariate regression analysis*

As shown in Tables 3 and 4, the statistical significance and effect size of the association between vitamin B6 and vitamin B12 intake and lung function indices (FEV_1_ and FVC) were assessed by adjusting 3 different models (Model I, Model II, Model III) to control for potential confounders. The results showed that vitamin B6 and vitamin B12 intake were positively associated with both FEV_1_ and FVC; that is, higher intake was associated with higher lung function indices. Specifically, for each 1 mg/day increase in vitamin B6 intake, FVC increased by 37 mL in Model I (*P* < .001), 31 mL in Model II (*P* < .001), and 22 mL in Model III (*P* = .004). For each 1 μmg/day increase in vitamin B12 intake, FEV_1_ increased by 2.6 mL (*P* = .027) in Model I, 2.4 mL (*P* = .009) in Model II, and 2.2 mL (*P* = .006) in Model III; FVC increased by 4.7 mL (*P* = .002) in Model I, 4.2 mL (*P* = .002) in Model II, and 3.8 mL in Model III (*P* < .001). In addition, when vitamin B6 intake was higher than 1.7 mg/day, FEV_1_ and FVC were significantly increased in Model I and Model II, but the effect was attenuated in Model III, and when vitamin B12 intake was higher than 2.4 μmg/day, FEV_1_ and FVC were significantly increased in all models. Overall, vitamin B6 and vitamin B12 intake showed a positive association with lung function, but this effect was attenuated when more confounding factors were taken into account, suggesting that other factors (e.g., lifestyle and health status) may also have a significant impact on lung function.^[17]^

**Table 3:** Multivariable linear regression analyses of vitamin B6 and B12 intake in relation to FEV_1_.

	FEV_1_, mL					
	Adjusted Model I		Adjusted Model II		Adjusted Model III	
	Adjusted β (95% CI)	*P*	Adjusted β (95% CI)	*P*	Adjusted β (95% CI)	*P*
Vitamin B6 (mg/d)	22 (9.7, 35)	**<.001**	16 (3.9, 28)	**.011**	11 (−1.8, 24)	.088
Below 1.7	Ref		Ref		Ref	
Above 1.7	65 (25, 105)	**.002**	46 (5.1, 87)	**.029**	34 (−9.0, 78)	.110
Vitamin B12 (μmg/d)	2.6 (0.31, 4.9)	**.027**	2.4 (0.64, 4.2)	**.009**	2.2 (0.69, 3.7)	**.006**
Below 2.4	Ref		Ref		Ref	
Above 2.4	67 (33, 100)	**<.001**	63 (24, 102)	**.003**	55 (17, 92)	**.006**

Crude: unadjusted.

Model I: adjusted for sex, age, race, BMI, poverty-income ratio, and education level.

Model II: adjusted for all covariates in Model I plus smoking status and alcohol drinking frequency.

Model III: adjusted for all covariates in Model II plus diabetes, total dietary fiber intake, urinary albumin, and urinary creatinine.

Bold values indicate statistically significant differences (*P* < .05).

BMI = body mass index, CI = confidence interval, FEV_1_ = forced expiratory volume in the first second, FVC = forced vital capacity.

**Table 4:** Multivariable linear regression analyses of vitamin B6 and B12 intake in relation to FVC.

	FVC, mL					
	Adjusted Model I		Adjusted Model II		Adjusted Model III	
	Adjusted β (95% CI)	*P*	Adjusted β (95% CI)	*P*	Adjusted β (95% CI)	*P*
Vitamin B6 (mg/d)	37 (25, 50)	**<.001**	31 (18, 43)	**<.001**	22 (7.7, 36)	**.004**
Below 1.7	Ref		Ref		Ref	
Above 1.7	90 (52, 128)	**<.001**	76 (31, 120)	**.002**	50 (−4.2, 105)	.069
Vitamin B12 (μmg/d)	4.7 (1.9, 7.4)	**.002**	4.2 (1.7, 6.8)	**.002**	3.8 (1.7, 5.9)	**<.001**
Below 2.4	Ref		Ref		Ref	
Above 2.4	84 (42, 125)	**<.001**	84 (40, 129)	**<.001**	70 (26, 115)	**.003**

Crude: unadjusted.

Model I: adjusted for sex, age, race, BMI, poverty-income ratio, and education level.

Model II: adjusted for all covariates in Model I plus smoking status and alcohol drinking frequency.

Model III: adjusted for all covariates in Model II plus diabetes, total dietary fiber intake, urinary albumin, and urinary creatinine.

Bold values indicate statistically significant differences (*P* < .05).

BMI = body mass index, CI = confidence interval, FVC = forced vital capacity.

### 3.5. Sex-specific differences in the association of vitamin B6 and B12 intake with lung function

In addition, recent literature suggests that diet may affect lung function differently according to gender,^[18]^ and we further explored the role of gender in the effect of vitamin B6 and vitamin B12 intake on lung function. As the results in Table 5 show, in stratified analyses by sex (**based on fully adjusted Model III**), we observed a positive association between vitamin B6 and B12 intake and lung function in both men and women. Based on point estimates, the association strength appeared higher in men (e.g., FEV_1_ increment in the high vitamin B6 intake group: 212 mL in men vs 84 mL in women). However, formal interaction tests did not reach statistical significance. Therefore, the data from this study are insufficient to conclude that there are clear gender differences in the effects of vitamins on lung function. The observed numerical differences may require validation in studies with larger sample sizes.

**Table 5:** Sex-stratified associations between vitamin intake and lung function (fully adjusted Model III).

	FEV_1_, mL			
	Sex adjusted male		Sex adjusted female	
	Adjusted β (95% CI)	*P*	Adjusted β (95% CI)	*P*
Vitamin B6 (mg/d)	60 (42, 77)	**<.001**	**39** (18**, 60**)	**<.001**
Below 1.7	Ref		Ref	
Above 1.7	212 (138, 286)	**<.001**	84 (26, 142)	**.005**
Vitamin B12 (μmg/d)	4.0 (−2.4, 10)	.200	2.3 (−2.8, 7.4)	.400
Below 1.7	Ref		Ref	
Above 1.7	207 (122, 291)	**<.001**	88 (40, 136)	**<.001**

Bold values indicate statistically significant differences (*P* < .05).

CI = confidence interval, FEV_1_ = forced expiratory volume in the first second.

### 3.6. The relationship between vitamin B6 and vitamin B12 intake and the risk of developing ventilatory impairment

We further explored the association between vitamin B6/B12 intake and the risk of ventilatory impairment (FEV_1_/FVC < 0.70), a threshold also used for chronic obstructive pulmonary disease diagnosis.^[19]^ As the results in Table 6 show, no significant association was found between vitamin B6 intake and airway obstruction, and the adjusted odds ratio was close to 1 in all models, with *P*-values >.05. For vitamin B12, although it showed some association in the unadjusted (Crude) model, in the progressively adjusted models (Model I, Model II, and Model III), its association with airway obstruction weakened. In stratified analyses, neither the high vitamin B6 nor vitamin B12 intake groups showed significant protection or increased risk. Overall, there was no significant association between vitamin B6 and airway obstruction, whereas the relationship between vitamin B12 and airway obstruction is unclear, and further studies are needed to determine its potential impact.

**Table 6:** Association between vitamin B6 and B12 intake and the risk of airway obstruction (FEV_1_/FVC < 0.70).

	Crude		Model I		Model II		Model III	
	Adjusted OR (95% CI)	*P*	Adjusted OR (95% CI)	*P*	Adjusted OR (95% CI)	*P*	Adjusted OR (95% CI)	*P*
Vitamin B6 (mg/d)	1.01 (0.93, 1.09)	.800	1.02 (0.93, 1.12)	.700	1.03 (0.94, 1.13)	.400	1.01 (0.94, 1.14)	.500
Below 1.7	Ref		Ref		Ref		Ref	
Above 1.7	1.00 (0.84, 1.21)	>.900	0.97 (0.78, 1.20)	.800	1.02 (0.84, 1.23)	.900	1.01 (0.82, 1.25)	>.900
Vitamin B12 (μmg/d)	1.01 (1.00, 1.02)	**.008**	1.01 (1.00, 1.02)	.085	1.01 (1.00, 1.02)	.150	1.01 (1.00, 1.02)	.010
Below 2.4	Ref		Ref		Ref		Ref	
Above 2.4	1.05 (0.89, 1.23)	.600	0.94 (0.78, 1.13)	.500	0.94 (0.76, 1.16)	.600	0.96 (0.78, 1.17)	.700

Crude: unadjusted.

Model I: adjusted for sex, age, race, BMI, poverty-income ratio, and education level.

Model II: adjusted for all covariates in Model I plus smoking status and alcohol drinking frequency.

Model III: adjusted for all covariates in Model II plus diabetes, total dietary fiber intake, urinary albumin, and urinary creatinine.

Bold values indicate statistically significant differences (*P* < .05).

BMI = body mass index, CI = confidence interval, FEV_1_ = forced expiratory volume in the first second, FVC = forced vital capacity, OR = odds ratio.

### 3.7. Nonlinear association of vitamin B6 and B12 intake with lung function

**Figure 2.**
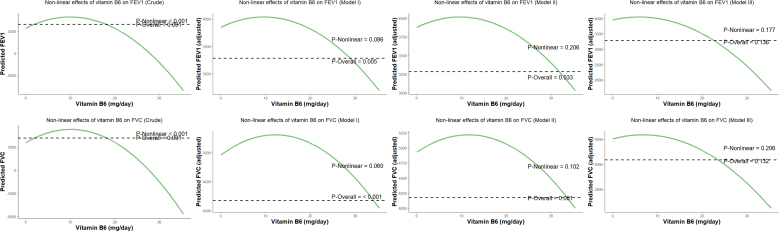
Association of vitamin B6 (mg/day) intake with lung function using restricted cubic spline regression. Nonlinear effects are not statistically significant after full adjustment. FEV_1_ = forced expiratory volume in the first second, FVC = forced vital capacity.

**Figure 3.**
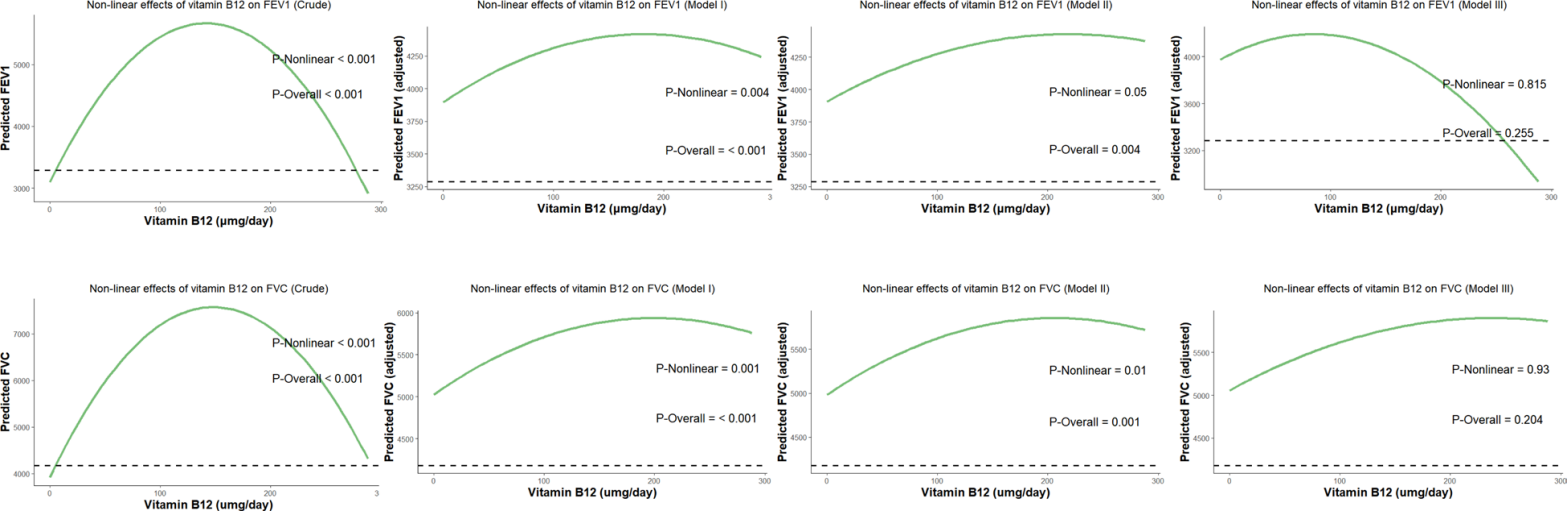
Association of vitamin B12 (μmg/day) intake with lung function using restricted cubic spline regression. Nonlinear effects are not statistically significant after full adjustment. Crude: unadjusted. Model I: adjusted for sex, age, race, BMI, poverty-income ratio, and education level; Model II: adjusted for all covariates in Model I plus smoking status and alcohol drinking frequency; Model III: adjusted for all covariates in Model II plus diabetes, total dietary fiber intake, urinary albumin, and urinary creatinine. BMI = body mass index, FEV_1_ = forced expiratory volume in the first second, FVC = forced vital capacity.

We used RCS regression to depict and evaluate the potential nonlinear relationships of vitamin B6 and vitamin B12 intakes with lung function indices (FEV_1_ and FVC). Each plot corresponds to a successive level of covariate adjustment, ranging from an unadjusted model (Crude) to models that incrementally added confounders (Model I–III). In the unadjusted analyses, vitamin B6 showed a statistically significant nonlinear association with lung function; after stepwise adjustment for sex, age, BMI, smoking, alcohol consumption, and other factors, FVC exhibited a significant overall positive trend, yet the nonlinear component remained nonsignificant, whereas the association with FEV_1_ attenuated after full adjustment. These findings suggest a modest beneficial effect of vitamin B6 on lung function that follows a largely linear trend, rather than a threshold. For vitamin B12, both FEV_1_ and FVC displayed significant nonlinear and overall trends in the crude models; however, sequential adjustment for sex, age, BMI, smoking, alcohol use, diabetes, and dietary factors progressively weakened these associations, with the nonlinear effect disappearing completely in Model III (*P*-nonlinear > .8). It should be noted that the curves depict associations across the observed range of intake. The tails of the curves (e.g., vitamin B6 > ~10 mg/day and vitamin B12 > ~50 µg/day) extend into intake levels that are uncommon through diet alone and are primarily based on extrapolation from a small number of high intake individuals; therefore, caution is warranted when interpreting the shape of the curve in these extreme ranges. The dashed lines represent the 95% confidence intervals, as shown in Figures 2 and 3.

## 4. Discussion

This study found that higher dietary intakes of vitamins B6 and B12 were associated with higher levels of specific lung function indices in a relatively healthy US population without cancer, asthma, chronic bronchitis, or emphysema. Specifically, higher dietary intake of vitamin B12 was independently correlated with FEV_1_ and FVC, whereas higher vitamin B6 intake was independently correlated with FVC. RCS analyses showed that the nonlinear effect of vitamin B6 on FVC was not significant, with no clear threshold or plateau observed; the effect size diminished after full covariate adjustment; the initially prominent nonlinear curves for vitamin B12 with both FEV_1_ and FVC gradually “flattened” as more confounders were added and completely disappeared in Model III (*P*-nonlinear > .8). This pattern “linearity remains, nonlinearity vanishes” suggests that the pulmonary protection signal for B6 warrants further validation in populations with different intake levels, whereas the previously reported “inverted-U” association for B12 is more likely attributable to residual confounding from uncontrolled dietary and lifestyle factors. Additionally, the RCS analysis in this study revealed that at extremely high intake levels (e.g., vitamin B6 > 25 mg/day), the curve tended to flatten or exhibit fluctuations (Fig. 2). It should be noted that these extreme ranges represent only a minority of individuals in the population who use high-dose supplements. The sparseness of data in these ranges increases statistical uncertainty (reflected in the widening of confidence intervals). Therefore, the shape of the curve at its extremes should not be overinterpreted. The primary conclusion of this study that vitamin intake exhibits a positive, broadly linear association with lung function within physiologically relevant and common intake ranges (e.g., vitamin B6 at 1–10 mg/day, vitamin B12 at 2–50 μg/day) is supported by more reliable data. Future intervention studies should focus on validating these associations within safe and achievable dietary and supplemental intake ranges. In summary, vitamins B6 and B12 may have potential positive associations with lung health, and higher dietary intake may be associated with better lung function in healthy individuals. However, because of the cross-sectionaldesign of this study, the need for further prospective and interventional studies was emphasized to validate these findings and to inform the development of nutritional guidelines. After all, maintaining good lung function is essential to living a healthy life,^[20]^ and the key strategy for preventing chronic respiratory disease remains quitting smoking.^[21]^

This study observed a positive correlation between vitamin B12 and lung function in the general population, differing from perspectives in some studies focused on pulmonary diseases. For instance, although a large prospective cohort study found no significant association between serum vitamin B12 and lung cancer risk,^[22]^ the role of B vitamins in maintaining DNA integrity and regulating gene expression through one-carbon metabolism has been extensively discussed.^[23]^ Notably, vitamin B12 is frequently used in lung cancer clinical treatment to mitigate the toxicity of pemetrexed chemotherapy,^[10]^ suggesting its critical role in cellular metabolism. These studies indicate that vitamin B12 may indirectly influence lung tissue by affecting fundamental biological processes such as cellular metabolism and repair. This potentially provides a mechanistic clue for the association with lung function observed in this study.

The present study found a significant positive correlation between vitamin B12 and lung function, a result that differs from the findings of Johansson et al.^[22]^ The present study focused on the correlation between vitamins B6 and B12 and lung function using a cross-sectional study design that focused on a relatively healthy population, excluding individuals with cancer, asthma, chronic bronchitis, or emphysema. In contrast, the Johansson et al^[22]^ study was a prospective cohort study in which the level of risk of disease was assessed by long-term follow-up, and although this approach can provide valuable information about temporal sequence, it may present challenges in controlling for confounding factors. This difference in study design and population selection may be an important reason for the inconsistent results.

The observed positive associations of vitamin B6 with lung function may be partly explained by its anti-inflammatory and antioxidant properties. As a key cofactor in numerous enzymatic reactions, particularly as pyridoxal phosphate (PLP)^[24]^ – vitamin B6 plays a vital role in regulating immunity and oxidative stress. Not only does it have anti-inflammatory and antioxidant properties against airway hyperresponsiveness,^[25]^ but in an animal model of microbial sepsis, vitamin B6 had a significant modulating effect on inflammatory and oxidative stress markers in rat lungs. Vitamin B6 not only reduced neutrophil infiltration in the organs and lowered the levels of oxidative markers, but also restored catalase activity in the lungs of septic animals. This suggests that vitamin B6 has significant anti-inflammatory and antioxidant effects in the lungs following microbial sepsis.^[26]^ Cheng et al’s^[27]^ study of chronic obstructive pulmonary disease patients showed that low vitamin B6 intake was significantly associated with an increased risk of frailty, while other B vitamins did not show similar associations. In addition, Shakoor et al found that vitamin B6 was able to inhibit cytokine storm and inflammatory responses in (COVID-19) patients by increasing levels of the anti-inflammatory cytokine interleukin‑10.^[28]^ Interleukin‑10 is a potent anti-inflammatory and immunosuppressive factor that inactivates macrophages and monocytes and inhibits the activity of antigen-presenting cells and T cells. Patients with COVID-19 typically exhibit T-lymphocyte overreactivity and overproduction of proinflammatory cytokines, and the modulatory effects of vitamin B6 may help to mitigate this excessive immune response.^[29]^ The anti-inflammatory and antioxidant properties that vitamin B6 possesses in lung tissue may provide a more in-depth elucidation of the significant associations between increased intake of vitamin B6 and improvement in lung function parameters that we found in our study. However, there are numerous other studies on vitamin B6. It is also useful for diabetes,^[30]^ cardiovascular disease,^[31]^ and cancer.^[32]^ A wide range of chronic diseases has potential preventive and palliative effects. Zemel et al’s study demonstrated that obese individuals who received a 4-week nutritional combination providing 30 mg PLP plus leucine exhibited a 20% drop in the oxidative stress marker malondialdehyde, alongside improved insulin sensitivity and enhanced lipid metabolism.^[33]^

Vitamin B12 (cobalamin) deficiency is relatively common in specific populations such as vegetarians.^[34–36]^ Its significance lies in its role as a coenzyme for methionine synthase and methylmalonyl-coenzyme A decarboxylase, participating in one-carbon metabolism and energy metabolism. The positive association with lung function identified in this study may relate to its role in maintaining normal cellular function, including that of lung tissue cells, through these metabolic pathways. Zhang et al^[37]^ first explored the role of vitamin B12 levels and metabolic gene variants in the pathogenesis of tuberculosis and finally selected 10 single-nucleotide polymorphisms for the associations between vitamin B12 metabolism-related genes (*TCN1*, *TCN2*, *CUBN*, *MMACHC*, *FUT6*, and *MUT*) and susceptibility to TB, and in TB patients. Plasma vitamin B12 levels were significantly lower in TB patients than in normal controls, and it is hypothesized that vitamin B12 and its metabolic gene variants may be closely related to the pathogenesis of TB and affect susceptibility. Bucca et al^[38]^ showed that vitamin B12 (cobalamin) deficiency can cause damage to the central nervous system and peripheral nervous system. This deficiency might induce sensory neuropathy and thus play a role in the pathogenesis of chronic cough. In patients with unexplained cough and laryngeal hyperresponsiveness, supplementation with cobalamin often resulted in significant relief of the associated symptoms, strongly suggesting a key role for vitamin B12. In addition, Arsenault et al found that consumption of nutritious snacks rich in vitamin B12 was effective in reducing the incidence of coughing,^[39]^ which further implies that vitamin B12 deficiency (Cbl-D) may influence the onset of coughing by exacerbating sensory neuropathy. The indispensability of vitamin B12 in human physiological functions may explain, in part, the significant association between higher vitamin B12 intake and improvement in pulmonary function parameters observed in our study. Vitamin B12 deficiency has also been closely linked to a number of diseases, including neural tube defects,^[40]^ cardiovascular disease,^[41]^ cognitive decline,^[42]^ macrocytic anemia,^[43]^ and osteoporosis,^[44,45]^ among others. However, excessive intake of vitamin B12 may have adverse effects. A previous study^[46]^ found that excessive vitamin B12 intake was associated with adverse outcomes in COVID-19 pneumonia.

The roles of vitamins B6 and B12 in modulating inflammation, oxidative stress, and cellular metabolism across diverse diseases highlight their fundamental biological functions. These general mechanisms provide a plausible framework for understanding their potential association with lung tissue health and function, as observed in our study, although direct pulmonary evidence remains to be fully established.

The association between vitamin B6/B12 intake and lung function suggests potential mechanisms involving one-carbon metabolism and oxidative stress regulation. However, most current evidence is derived from in vitro studies or non-pulmonary tissues such as the liver and brain, highlighting an urgent need for lung-specific research validation. Existing experimental studies indicate that vitamin B6 can elevate glutathione levels and reduce lipid peroxidation via the transsulfuration pathway (homocysteine → cystathionine → cysteine).^[26]^ Additionally, in the form of PLP, it inhibits the NLRP3 inflammasome, thereby decreasing the release of interleukin‑1 beta, interleukin‑6, and tumor necrosis factor alpha.^[47]^ If these effects similarly occur in the lungs, they could theoretically alleviate airway oxidative damage and chronic inflammation. Moreover, through one-carbon units provided by PLP, vitamin B6 may promote the proliferation of type II alveolar epithelial cells and the renewal of surfactant, thereby helping to maintain small airway patency and lung compliance. However, to date, no studies have directly quantified these pathways in lung tissue or at the level of pulmonary function. As for vitamin B12, it serves as an essential coenzyme for methionine synthase, catalyzing the conversion of homocysteine to methionine to sustain the production of S-adenosylmethionine, the universal methyl donor. S-adenosylmethionine-dependent methylation is critical for the posttranslational modification of elastin, collagen, and surfactant proteins,^[48]^ though much of the supporting evidence comes from neural and vascular tissues.^[49]^ Furthermore, vitamin B12 acts as a coenzyme for methylmalonyl-coenzyme A mutase. A deficiency in B12 leads to elevated methylmalonic acid (MMA), which can inhibit mitochondrial complex I and increase reactive oxygen species, thereby inducing chronic low-grade inflammation – a mechanism confirmed in rat liver models and human serum studies.^[50]^ If a similar biochemical cascade occurs in the lungs, adequate B12 levels may help preserve lung elastic recoil and small airway stability by ensuring proper matrix protein methylation and suppressing the MMA-reactive oxygen species axis, thereby improving airflow limitation.^[51]^ However, whether a critical MMA concentration is reached in the lungs and whether such a concentration significantly affects FEV_1_/FVC remain to be clarified through prospective or interventional studies.

This study holds significant implications for clinical and public health nutrition. High intake of vitamins B6 and B12 is associated with better lung function in healthy US adults (without chronic respiratory disease), potentially contributing to lung health maintenance and enhancement. This underscores the importance of consuming these vitamins and suggests healthcare professionals incorporate nutritional counseling into respiratory health promotion strategies. However, while statistically significant associations between vitamin B6/B12 intake and lung function were observed, it is important to note the modest absolute effect sizes (e.g., a 1 mg/day increase in vitamin B6 may increase FVC by approximately 22 mL). Compared to normal inter-individual variability in lung function, such gains are limited and may lack significant short-term clinical relevance at the individual level. However, from a public health and prevention perspective, long-term maintenance of adequate vitamin intake may yield cumulative benefits by slowing age-related lung function decline, particularly affecting subgroups with insufficient intake. Future long-term follow-up studies are needed to validate this hypothesis and clarify clinical significance for specific populations.

### 4.1. Strengths and limitations

The NHANES database was chosen as the data source for this study, which used a complex stratified multistage sampling design that is representative of the health and nutritional status of the entire US population. Its data cover a wide range of dimensions, including demographics, dietary intake, physical examination, laboratory test results, and lifestyle, which provided rich statistical information for this study. However, there are some limitations of this study that may have an impact on the interpretation and application of the findings. First, given the cross-sectional nature of NHANES, this study can only indicate a statistical association between vitamin B6/B12 intake and lung function parameters and is insufficient to establish a causal relationship. Second, vitamin B6 and B12 intake was assessed by a 24-hour dietary recall interview, a method that may be subject to memory bias and subjective reporting, which may affect the veracity of the data. Third, due to a high proportion of missing values, physical activity was excluded from the analysis as it could influence spirometric outcomes. Fourth, the NHANES database does not include participant-specific occupational information; the absence of this key potential confounder affecting lung function undoubtedly limited the comprehensiveness and depth of the study. At the same time, the lack of long-term follow-up data prevented us from tracking dynamic changes in spirometry results, which limited our ability to study the long-term evolution of lung function. Fifth, there is a discussion on statistical methods and residual confounding. This study employed stepwise regression to construct a multivariate model, aiming to identify factors independently associated with lung function from a series of potential covariates. We acknowledge that while this approach facilitates model parsimony, its results may be influenced by variable entry/elimination criteria and carry the risk of omitting important confounders correlated with both exposure and outcome. Although we demonstrated the robustness of associations by presenting results across different adjustment levels (Crude, Model I–III), residual confounding cannot be entirely ruled out. Sixth, regarding sample representativeness and selection bias, for quality assurance, the study required participants to have complete data on lung function, diet, and key covariates, resulting in a final sample (n = 9648) smaller than the initially eligible NHANES population. Individuals with complete data may differ in health awareness or behavior. Thus, even after applying complex sampling weights (WTDRD1, the masked variance pseudo-stratum, the masked variance pseudo-primary sampling unit) for adjustment, the findings may not fully represent all US adults. The conclusions are mainly applicable to adults with evaluable data. However, given the biological plausibility of the associations and the robustness of the results across multiple models, such bias is unlikely to completely reverse the direction of the main findings.

## 5. Conclusion

In summary, this study examined the relationship between vitamin B6 and B12 intake and lung function based on nationally representative NHANES data from 3 consecutive cycles (2007–2012). The findings provide a novel framework for understanding the association between vitamin B6 and B12 intake and lung function, laying an important foundation for follow-up studies and providing a valuable reference for strategies to prevent and maintain lung function through dietary interventions. However, additional prospective studies or randomized controlled trials are still needed to clarify causality and further validate the potential associations of these vitamins with lung health.

## Acknowledgments

The authors thank all those involved in this cross-sectional study.

## Author contributions

**Conceptualization:** Wuzhen Wang.

**Data curation:** Wuzhen Wang.

**Formal analysis:** Wuzhen Wang.

**Investigation:** Wuzhen Wang.

**Methodology:** Yingying Gu.

**Project administration:** Yingying Gu.

**Resources:** Yingying Gu.

**Software:** Wuzhen Wang.

**Supervision:** Yingying Gu.
